# Re-organization of the Medical Department of Transylvania University

**Published:** 1837

**Authors:** 


### Art. III.—Re-organization of the Medical Department of Transylvania University.

Under our analytical head we gave an account of the disintegration of the Lexington School, and stated that Professors Dudley, Richardson and Short had been re-elected, Dr. Bush being appointed adjunct to Dr. Dudley. At the same time, Dr. James C. Cross, of the *Medical College of Ohio*, a native of the vicinity of Lexington, was chosen to the Chair, lately held by Dr. Caldwell, and accepted it. Subsequently, Dr. John Eberle, of the Ohio School, and Dr. Thomas D. Mitchell, formerly one of its professors, were elected to the Chairs of the Theory and Practice, and of Chemistry. They have accepted those stations, and the faculty is, therefore, re-organized out of materials that promise to reproduce and perpetuate its usefulness. D.

